# CORRELATES OF BREAST CANCER SCREENING BEHAVIORS AMONG INDIGENOUS WOMEN

**DOI:** 10.1093/geroni/igz038.923

**Published:** 2019-11-08

**Authors:** Soonhee Roh, Yeon-Shim Lee, Kyoung Hag Lee, Jung Sim Jun

**Affiliations:** 1 University of South Dakota, Sioux Falls, South Dakota, United States; 2 San Francisco State University, San Francisco, California, United States; 3 Wichita State University, Wichita, Kansas, United States; 4 Kansas State University, MANHATTAN, Kansas, United States

## Abstract

Cervical cancer remains a significant cause of morbidity and mortality among women globally; yet cancer burden is unevenly distributed among racial/ethnic groups. With 12,820 new cases in 2017 in the U.S., cervical cancer is the top cause of death among Indigenous women. Indeed, cervical cancer mortality rates among AI women in South Dakota are five times the national average and 79% higher compared to Whites in that region. This study examined predictive models of utilization of mammograms among Indigenous women adapting Andersen’s behavioral model. Using a sample of 285 Indigenous women residing in South Dakota, nested logistic regression analyses were conducted to assess predisposing (age and marital status), need (personal and family cancer history), and enabling factors (education, monthly household income, mammogram screening awareness, breast cancer knowledge, self-rated health, and cultural practice to breast cancer screening). Results indicated that only 55.5% of participants reported having had a breast cancer screening within the past 2 years, whereas 21.0% never had a mammogram test. After controlling for predisposing and need factors, higher education, greater awareness of mammogram, and higher utilization of traditional Native American approaches were significant predictors of mammogram uptake. The results provide important implications for intervention strategies aimed at improving breast cancer screening and service use among Indigenous women. Educating health professionals and Indigenous community members about the importance of breast cancer screening is highly needed. It is critical to assess a woman’s level of traditional beliefs and practices and its possible influence on screening participation and future screening intention.

